# Electronic Transport Properties in GaAs/AlGaAs and InSe/InP Finite Superlattices under the Effect of a Non-Resonant Intense Laser Field and Considering Geometric Modifications

**DOI:** 10.3390/ijms23095169

**Published:** 2022-05-05

**Authors:** John A. Gil-Corrales, Alvaro L. Morales, Melike Behiye Yücel, Esin Kasapoglu, Carlos A. Duque

**Affiliations:** 1 Grupo de Materia Condensada-UdeA, Instituto de Física, Facultad de Ciencias Exactas y Naturales, Universidad de Antioquia UdeA, Calle 70 No. 52-21, Medellín 050010, Colombia; jalexander.gil@udea.edu.co (J.A.G.-C.); alvaro.morales@udea.edu.co (A.L.M.); 2 Department of Physics, Science Faculty, Akdeniz University, 07058 Antalya, Turkey; myucel@akdeniz.edu.tr; 3 Department of Physics, Science Faculty, Sivas Cumhuriyet University, 58140 Sivas, Turkey; ekasap@cumhuriyet.edu.tr

**Keywords:** GaAs/AlGaAs–InSe/InP superlattice, transmission probability, Landauer formalism, intense laser field

## Abstract

In this work, a finite periodic superlattice is studied, analyzing the probability of electronic transmission for two types of semiconductor heterostructures, GaAs/AlGaAs and InSe/InP. The changes in the maxima of the quasistationary states for both materials are discussed, making variations in the number of periods of the superlattice and its shape by means of geometric parameters. The effect of a non-resonant intense laser field has been included in the system to analyze the changes in the electronic transport properties by means of the Landauer formalism. It is found that the highest tunneling current is given for the GaAs-based compared to the InSe-based system and that the intense laser field improves the current–voltage characteristics generating higher current peaks, maintaining a negative differential resistance (NDR) effect, both with and without laser field for both materials and this fact allows to tune the magnitude of the current peak with the external field and therefore extend the range of operation for multiple applications. Finally, the power of the system is discussed for different bias voltages as a function of the chemical potential.

## 1. Introduction

The study of semiconductor systems has advanced significantly during the last fifty years, particularly in systems based on superlattices (SLs) of GaAs, InSe, among other materials. Modern experimental techniques have allowed us to understand the behavior of charge carriers within these heterostructures in a much more precise way, and theoretical models with a high degree of precision have been developed hand in hand with theoretical models. A significant motivation for the study of heterostructures based on these materials is the novel applications in fields such as the development of field-effect transistors or high electronic mobility, systems in which the effects of impurities, pressure, and applied fields have been analyzed to improve optical absorption; these devices are candidates for the advancement of future technologies in the electronics field [1,2,3,4,5]. Likewise, in this field, there are switching devices capable of working at high speed [6]. In the optoelectronics field, the development of solar cells that can provide strong absorption in a much wider range of the electromagnetic spectrum stands out, which considerably improves the efficiency of usable energy [7,8,9,10,11,12,13,14,15,16]. A typical application of semiconductor systems is in detectors, which, depending on the type of material or the geometric arrangement, can be tuned to detect ultraviolet, infrared, and even up to the terahertz range [17,18,19,20,21,22,23]. In this field, it is also necessary to mention the cascade laser [24]. Finally, various applications such as biosensors to detect some types of cancer cells or Zeno-logical applications [25,26] are worth mentioning. In some of these applications, low-dimensional semiconductor devices such as quantum dots, quantum wires, or quantum wells are implemented [27,28].

Some of the first studies on SLs based on semiconductor systems were developed around the 1970s in works such as those by Esaki and Chang [29] in which the properties of electronic transport in systems with a periodic structure of GaAs/AlAs were experimentally analyzed. By means of the molecular-beam epitaxy technique, in their work, the evidence of the system oscillatory conductance behavior increasing voltage was found. Later, using the same growth technique for the heterostructures, Dingle et al. [30] experimentally demonstrated the formation of a GaAs/AlGaAs SL at low temperatures using optical-absorption measurements on ultra-thin layers. Later, in the 1990s, Fedirko and Eremtchenko [31] analyzed the SLs based on GaAs/AlGaAs by means of scanning probe microscopy, finding a pretty good periodic structure.

In more recent years, the SLs of semiconductor materials continue to be studied either to understand excitations in the system or to analyze the response under external fields. In works such as that of Komatsu et al. [32], the authors analyzed the intensity of exciton photoluminescence in the presence of an external magnetic field in a GaAs SL. In this type of material, the study of structural properties is of great importance, since it is possible to tune electronic properties that lead to the optical response of the system. In 2004, Jeong et al. [33] studied these properties in a GaAs/AlGaAs SL layer on InAs quantum dots by means of photoluminescence, photoreflectance spectroscopy, and transmission electron microscopy. Their results showed that the wavelength of the quantum dots was effectively tailored by the high potential barriers. The effect of the interface on the modulation-doped of the SL-type heterostructures can considerably modify the electronic properties in *n*-doped and *p*-doped systems. This effect was studied by Bezerra et al. [34], finding that the presence of graded interfaces modifies the carrier confinement inside of the GaAs quantum well. When the semiconductor system is subjected to the action of an external magnetic field, it is possible to modify the properties of electronic transport, in particular, the current–voltaget-voltage curves, or to generate the appearance of Magnetoresistance oscillations [35,36].

Currently, there are numerous experimental works on SL systems grown mainly using methods such as molecular beam epitaxy (MBE) that lead to potential applications. To mention some of these works, in 2021, Kesaria et al. [37] developed an InAs/GaSb and InAs/InAsSb SL using MBE to improve performance and optimize NOx detection systems. Another of the common applications of SL systems based on semiconductor materials is high harmonic multipliers. This type of system can be optimized by means of the geometric modifications developed in this work. As a reference to this application the manuscripts of Apostolakis et al. [38] and Pereira et al. [39] that use a SL for harmonic multiplication in the Gigahertz–Terahertz range. To mention one last application, in 2022, Ting et al. [40] explored an InAs/InAsSb SL for potential NASA land-imaging applications.

One way to characterize the semiconductor structures is by analyzing the transport properties, whether thermal or electrical, depending on if the system is put in contact with electronic reservoirs at different temperatures or if the system is subjected to a potential difference. These properties can be analyzed by means of the Landauer–Buttiker formalism [41,42].

In this article, we are interested in studying the electronic transport properties of a finite periodic lattice. We are particularly interested in studying the tunneling current considering geometric variations of the heterostructure for two different combinations of the semiconductor materials, GaAs/AlGaAs and InSe/InP. The appearance of NDR in the current–voltage characteristics for all systems is discussed, which is of particularity high importance when it comes to practical applications in devices. It should be noted that the appearance of NDR zones is not exclusive to systems SLs; this behavior is also evidenced in molecular devices [43]. Using the Landauer formalism, the current due to the tunneling of electrons from the emitter to the collector, generated by a potential difference between the terminals of the device, is calculated. The power due to the diffusion of charge carriers and the effects of a non-resonant intense laser field (ILF) on the conduction band profile and, therefore, on the conduction current are also studied. The solution of the differential equations and the calculation of the electronic transmission probability have been carried out using the finite element method (FEM). The paper is organized as follows: Section 2 contains the theoretical framework; Section 3 is devoted to the results and corresponding discussion; finally, in Section 4, the main conclusions are presented.

## 2. Theoretical Model

**Figure 1:**
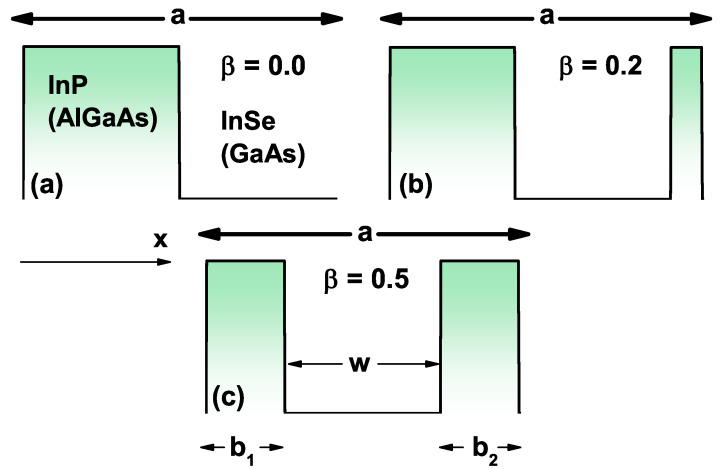
(**a**–**c**) Scheme corresponding to a single period of the superlattice for β=0.0, β=0.2, and β=0.5, respectively. These parameters are entered in Equations (1) and (2) and Table 1.

The system under study corresponds to a SL with InSe (GaAs) well regions and InP (AlGaAs) barrier regions. As shown in Figure 1, each well has a *w*-width and is made of InSe (GaAs) and the left and right barriers of the first period have $b_{1}$ and $b_{2}$ lengths, respectively, and are of InP (AlGaAs) material, as shown in Figure 1c. The full length of the first period is *a* and remains fixed for all three figures (see Figure 1a–c). The total length of the barriers inside the first cell is *b*. From the above explanations, it is possible to define the additional parameters, the γ-parameter that sets the relationship between the total barrier length and the length of the first SL period, and the β-parameter that can modify the geometry of the complete periodic system, with 0≤β≤0.5:

(1) $$\begin{array}{cc} a & =b_{1}+b_{2}+w\;,\\ b & =b_{1}+b_{2}\;,\\ \gamma & =b/a\;,\\ \beta & =b_{2}/b\;. \end{array}$$

As we see in Figure 1, depending on the β-value, the first SL period will have an established form: (i) for β=0.0, the barriers will have the same width as the wells; (ii) for β=0.2, the system will be asymmetric regardless of the number of periods, and the well on the right side will always be thinner than the one on the left side, and finally, (iii) for β=0.5, the system will be symmetric for all periods, but the two lateral barriers will always be smaller than the central barriers. Depending on the number of calculated periods and each β-value, Figure 1a–c will be repeated periodically.

From the above explanations, it is possible to write expressions for the barrier and well widths in terms of the γ, β, and *a* parameters, i.e.,

(2) $$\begin{array}{cc} b_{1} & =\gamma \;a\;(1-\beta )\;,\\ b_{2} & =\gamma \;a\;\beta \;,\\ w & =a\;(1-\gamma )\;. \end{array}$$

Table 1 shows the particular case with β = 0.0, 0.2, and 0.5,

The shape of each potential can be modified by means of variations in the geometric parameters according to Equation (2) and Table 1, to later be replaced in the time-independent Schrödinger equation to obtain a set of eigenfunctions and eigenvalues ($\psi_{n,i}^{\beta }\left(\vec{r}\right)$ and $E_{n,i}^{\beta }$),

(3) $$\left[\vec{\nabla }\cdot \left(\frac{-\hbar^{2}}{2\;m^{*}\left(x\right)}\vec{\nabla }\right)+U_{n}^{\beta }\left(x\right)\right]\psi_{n,i}^{\beta }\left(\vec{r}\right)=E_{n,i}^{\beta }\;\psi_{n,i}^{\beta }\left(\vec{r}\right)\;,$$

where $m^{*}\left(x\right)$ is the *x*-dependent electron effective mass (note that in this work, we deal with different values of the electron effective mass at the well and barrier regions). $U_{n}^{\beta }\left(x\right)$ is the SL-potential (the SL is grown along the *x*-direction), which depends directly on the *n*-parameter (number of SL periods, i.e., the number of times that each panel of Figure 1 is repeated for each β-parameter value). Additionally, $\psi_{n,i}^{\beta }\left(\vec{r}\right)$ is the electron wavefunction corresponding to the *i*-th quasi-stationary state and of course, it depends on both *n* and β. Finally, $E_{n,i}^{\beta }$ is the corresponding energy; in general, the eigenvalues have real and imaginary parts since the states are not stationary. Consequently, the electrons have a lifetime inside the wells to later leave these by quantum tunneling effect.

Using the separation of variables method, we can write $\psi_{n,i}^{\beta }\left(\vec{r}\right)=e^{i\;\vec{k_{⊥}}\cdot \vec{\rho }}\;\Psi_{n,i}^{\beta }\left(x\right)$, where $\vec{k_{⊥}}$ and $\vec{\rho }$ are the wavevector and electron coordinate along the yz-plane, perpendicular to the growth direction of the heterostructure. With the previous wavefunction inserted in Equation (3), and taking into account that we are dealing with the bottom of all energy sub-bands (meaning $\vec{k_{⊥}}=0$), we can obtain a 1D differential equation for the *x*-coordinate, whereby imposing the open boundary conditions, the $\Psi_{n,i}^{\beta }\left(x\right)$ function can be written as a linear combination of plane waves, as follows

(4) $$\Psi_{n,i}^{\beta }\left(x\right)=A_{n}^{\beta }\left(x\right)\;e^{+i\;k_{n,i}^{\beta }\;x}+B_{n}^{\beta }\left(x\right)\;e^{-i\;k_{n,i}^{\beta }\;x}\;.$$

The $A_{n}^{\beta }\left(x\right)$ and $B_{n}^{\beta }\left(x\right)$ functions are the probability amplitudes of the system. The first term on the right-hand side in Equation (4) corresponds to an electron moving from left to right with probability amplitude $A_{n}^{\beta }\left(x\right)$, whereas the second term is an electron moving from right to left (reflected wave) with probability amplitude $B_{n}^{\beta }\left(x\right)$; the complete wave function for any region of the system is a superposition of these plane waves. It is clear that these amplitudes must depend on the *x*-point, at which they are being calculated, as well as on the (*n*, β) geometric parameters of the SL. $k_{n,i}^{\beta }$ is the magnitude of the wave vector and is given by $k_{n,i}^{\beta }={\left(2m^{*}\left(E_{n,i}^{\beta }-U_{n}^{\beta }\left(x\right)\right)/\hbar^{2}\right)}^{1/2}$.

The *x*-dependent differential equation associated with the Equation (3) when the wave function represented by Equation (4) has been considered solved through the FEM with the COMSOL-Multiphysics licensed software (5.4, COMSOL AB, Stockholm, Sweden) [44,45,46] by implementing the semiconductor module (“Semiconductor Module User’s Guide” COMSOL Multiphysics^®^) [47,48,49,50]. In this way, the values of the probability amplitudes are found in any region of the system. After that, it is possible to calculate the electron transmission function $T_{n,i}^{\beta }\left(E\right)$ through the device as the quotient between the amplitude of the transmitted wave and the amplitude of the incident wave,

(5) $$T_{n}^{\beta }\left(E\right)=\frac{|A_{n}^{\beta }\left(x_{f}\right){|}^{2}}{|A_{n}^{\beta }\left(x_{i}\right){|}^{2}}\;,$$

where $A_{n}^{\beta }\left(x_{i}\right)$ represents the amplitude of the wave that propagates from left to right evaluated at the emitter and $A_{n}^{\beta }\left(x_{f}\right)$ is the amplitude of a wave that propagates from left to right but evaluated in the collector. This function is proportional to the probability of electron tunneling through the system.

Once the transmission probability has been calculated, it is possible to calculate the voltage–current characteristics in the SL using Landauer’s theory, which tells us that employing a connection of the system with two electronic reservoirs, it is possible to obtain the electronic tunneling current through the system, given by [51,52,53],

(6) $$I\left(\Lambda \right)=I_{0}\int_{-\infty }^{\infty }T_{n}^{\beta }\left(E,\Lambda \right)\left[f_{L}\left(E,\Lambda \right)-f_{R}\left(E,\Lambda \right)\right]dE\;,$$

where *e* is the electron charge, *ℏ* is the reduced Planck constant, and $I_{0}=e/\pi \hbar$. The terms $f_{L}\left(E,\Lambda \right)$ and $f_{R}\left(E,\Lambda \right)$ correspond to the Fermi functions evaluated at the emitter and collector, respectively. They are given by $f_{L}\left(E,\Lambda \right)={\left(1+e^{\left(E-E_{F}\right)/k_{B}T}\right)}^{-1}$ and $f_{R}\left(E,\Lambda \right)={\left(1+e^{\left(E-E_{F}+\Lambda \right)/k_{B}T}\right)}^{-1}$, where Λ is the bias voltage applied between both terminals of the device. By means of the transmission function, it is also possible to calculate the heat flux and the power in the system, given, respectively, by

(7) $$\begin{array}{c} \Theta_{n}^{\beta }\left(l\right)=\frac{2}{h}\int_{-\infty }^{\infty }T_{n}^{\beta }\left(E,\Lambda \right)\left(E-E_{f}\left(l\right)\right)\left(f_{L}\left(E,\Lambda \right)-f_{R}\left(E,\Lambda \right)\right)dE\;, \end{array}$$

and

(8) $$P_{n}^{\beta }=\Theta_{n}^{\beta }\left(L\right)-\Theta_{n}^{\beta }\left(R\right)\;.$$

The term $T_{n}^{\beta }\left(E,\Lambda \right)$ represents the transmission probability for a fixed voltage. $E_{f}\left(l\right)$ is the Fermi energy for l=L (l=R); that is, for the emitter (collector).

At this stage of our work, we must present some comments on the approaches and model that we have used. Our system corresponds to a SL with a small conduction region connected to two electronic reservoirs. When the confinement in this region is strong, the rigorous treatment of quantum effects becomes crucial. The Landauer formalism is a well-known method for describing these systems, which is based on an energy-dependent transmission probability for the region of interest. This method has been successfully applied to ballistic transport [54]. The effects of ballistic conduction are typically of 1D structures, in particular in SL semiconductors, because they present an extreme quantization effect. The small size of the SL (nanometer-scale) and the mean free path which can be longer than that for example in a metal, which allows neglecting the effects of impurities, geometric defects, thermal fluctuations of ions, etc. In other words, ballistic transport implies a problem without scattering. In the study by Gruss et al. [55], an extension of the Landauer formalism is presented to consider the electronic properties of transport; they developed an open system approach to transport that includes a finite electron lifetime representing the presence of these relaxation mechanisms. Additionally, in 2015, N. Sano [56] derived theoretical expressions of the impurity-limited resistance in the nanowire under the linear response regime from the Landauer formula and from the Boltzmann transport equation (BTE) with the relaxation time approximation. Currently, there are a variety of works in which the Landauer formalism is addressed, including impurity effects; that is, in a non-ballistic approach, some of these works are included in the Refs. [57,58].

In our study, we consider the effects of an *x*-polarized non-resonant intense laser field (ILF) applied to the SL structure, which can be modeled as a monochromatic plane wave with angular frequency ϕ. Due to the presence of this non-resonant laser field, a modification occurs in the potential profile that enters Equation (3). So, the transformation $U_{n}^{\beta }\left(x\right)\to \langle V_{n}^{\beta }\left(x,\alpha_{0}\right)\rangle$ is obtained through the relation

(9) $$\langle V_{n}^{\beta }\left(x,\alpha_{0}\right)\rangle =\frac{\phi }{2\pi }\;\int_{0}^{2\pi /\phi }U_{n}^{\beta }\left[x+\alpha_{0}\sin\left(\phi \;t\right)\right]\;dt\;.$$

The $\langle V_{n}^{\beta }\left(x,\alpha_{0}\right)\rangle$ potential is known as the laser-dressed potential [59,60], where the ILF-parameter is defined as $\alpha_{0}=\left(eA_{0}\right)/\left(m^{*}\phi \right)$ (here, $A_{0}$ is the strength of the laser field). Equation (9) is obtained by applying an intense, high frequency laser field to an atomic system [61,62,63]. The corresponding time-dependent Schrödinger equation has the form,

(10) $$\begin{array}{c} i\hbar \;\frac{\partial }{\partial t}\;\psi_{n,i}^{\beta }\left(\vec{r},t\right)=\left[\frac{{\left(\hat{\vec{p}}-e\;\vec{A}\left(\vec{r},t\right)\right)}^{2}}{2\;m^{*}\left(x\right)}+V\left(\vec{r}\right)\right]\psi_{n,i}^{\beta }\left(\vec{r},t\right)\;, \end{array}$$

where $\hat{\vec{p}}$ is the momentum operator, *e* is the electron charge, $\vec{A}\left(\vec{r},t\right)$ is the magnetic vector potential associated with the external laser field, and $V(\vec{r})$ is the confinement potential of the system. Using the Coulomb gauge, the dipole approximation for the magnetic vector potential, and the Kramers–Henneberger transformations [64], the last equation obtains the form,

(11) $$\begin{array}{c} i\hbar \;\frac{\partial }{\partial t}\;\psi_{n,i}^{\beta }\left(\vec{r},t\right)=\left[\vec{\nabla }\cdot \left(\frac{-\hbar^{2}}{2\;m^{*}\left(x\right)}\vec{\nabla }\right)+V\left(\vec{r}+\vec{\alpha }\left(t\right)\right)\right]\psi_{n,i}^{\beta }\left(\vec{r},t\right)\;, \end{array}$$

where $\vec{\alpha }\left(t\right)=\alpha_{0}\;\sin\left(\phi t\right)\;\hat{x}$, with $\hat{x}$ the system growth direction, and $\alpha_{0}$ is the ILF-parameter previously defined. Equation (11) indicates that the effect of the non-resonant ILF generates an effective displacement $\vec{\alpha }\left(t\right)$ on the system potential. Considering only a high-frequency laser field in Equation (11), performing a Fourier expansion and using Floquet theory [65] at the $V(\vec{r}+\vec{\alpha }\left(t\right))$ term, Equation (9) is obtained for the time-independent total potential. From the above, the time-independent Schrödinger equation is finally obtained, including the effect of the intense non-resonant laser field in the growth direction of the structure. In this way, the time-independent Schrödinger equation is derived, which includes the laser effect,

(12) $$\left[\vec{\nabla }\cdot \left(\frac{-\hbar^{2}}{2\;m^{*}\left(x\right)}\vec{\nabla }\right)+\langle V_{n}^{\beta }\left(x,\alpha_{0}\right)\rangle \right]\Psi_{n,i}^{\beta }\left(x\right)=E_{n,i}^{\beta }\;\Psi_{n,i}^{\beta }\left(x\right)\;.$$

## 3. Results and Discussion

For the calculations, the following input parameters were used at 300 K: $m^{*}=0.067\;m_{0}$ ($m^{*}=0.0879\;m_{0}$) for the GaAs (AlGaAs) electron effective mass (where $m_{0}$ is the mass of the free electron) and $U_{n}^{\beta }\left(x\right)=0$ ($U_{n}^{\beta }\left(x\right)=0.261$ eV) in the GaAs (AlGaAs) material [66,67]. Additionally, $m^{*}=0.023\;m_{0}$ ($m^{*}=0.08\;m_{0}$) is the InSe (InP) and $U_{n}^{\beta }\left(x\right)=0$ ($U_{n}^{\beta }\left(x\right)=0.57$ eV) in the InSe (InP) material [68]. Geometric parameters: unit cell length *a* = 10 nm, γ = 0.5, and angular laser frequency ϕ=1 THz. The equations were solved through the FEM considering the following parameters: 5000 elements, 2 edge elements, a 1.0 element-length radius, and 5001 mesh vertices.

**Figure 2:**
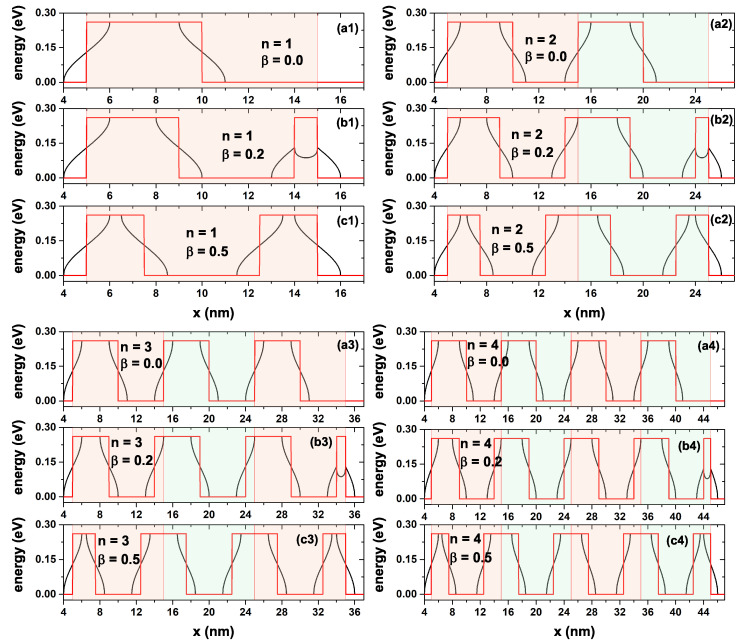
*x*-dependent potential profile of a GaAs-Al${}_{0.3}$Ga${}_{0.7}$As lattice varying from n=1 to n=4 periods. The shape of the periods is also modified with the β= 0.0, 0.2, and 0.5 parameters. In (**ai**), β = 0.0 and *n* = {1–4} for i = {1–4} respectively. In (**bi**), β = 0.2 and *n* = {1–4} for i = {1–4} respectively. In (**ci**), β = 0.5 and *n* = {1–4} for i = {1–4} respectively. The red solid lines indicate the potential with $\alpha_{0}=0$, whereas the black solid line shows the potential modified with $\alpha_{0}=1.0$ nm. The different-color shadow regions indicate the SL periods for every system.

Figure 2 shows the bottom of the conduction band for a GaAs-Al${}_{0.3}$Ga${}_{0.7}$As lattice (for the InSe-InP system, it would be an equivalent figure, only the value of the band offset changes) varying from one to four periods and three values of the β-parameters. The red solid lines indicate the potential without the ILF effect, whereas the black solid curved regions show the potential modified by an ILF-parameter of $\alpha_{0}=1.0$ nm. As we can see in Figure 2, when β=0, the number of barriers is equal to the *n*-parameter, whereas, for β≠0, the number of barriers is equal to n+1. Each region shaded with light blue or light red color corresponds to a SL period. Note that depending on the *n* and β values, in the union of two or more periods, an overlap of the barrier regions may occur; for example, for n=2 and β=0.5, which corresponds to Figure 2(c2), the union of two periods generates the appearance of a central barrier of 5 nm wide. This is wider than the two lateral barriers that each measure 2.5 nm. Note how applying an ILF to the system can significantly modify the shape of the potential barriers; this variation is more significant for the barriers of smaller width, as can be seen, for example, in Figure 2(b1–b4) for β=0.2; when comparing this effect on the right barrier with the other barriers, a clear decrease in the height of the barrier on the right is seen. This effect is not observed for β=0 and β=0.5.

**Figure 3:**
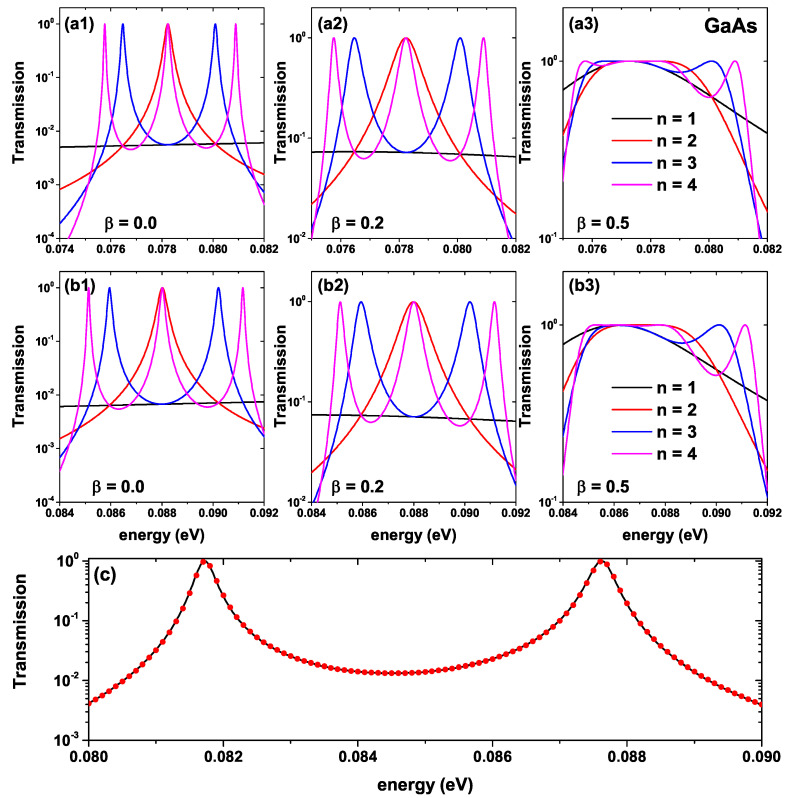
Energy dependence of the electronic transmission probabilities for a GaAs-Al${}_{0.3}$Ga${}_{0.7}$As finite lattice, according to Figure 2, i.e., varying the *n*-number of periods and the shape according to the structural β-parameter. Results are without ILF effects ((**a**) panels) and with $\alpha_{0}=1$ nm ((**b**) panels). In (**a1**), $\alpha_{0}=0$ nm and β = 0.0. In (**a2**), $\alpha_{0}=0$ nm and β = 0.2. In (**a3**), $\alpha_{0}=0$ nm and β = 0.5. In (**b1**), $\alpha_{0}=1$ nm and β = 0.0. In (**b2**), $\alpha_{0}=1$ nm and β = 0.2. In (**b3**), $\alpha_{0}=1$ nm and β = 0.5. (**c**) Comparison between the transfer matrix method and the FEM.

Figure 3 shows the energy dependence of the electronic transmission function for the GaAs-Al${}_{0.3}$Ga${}_{0.7}$ as-well-barrier system presented in Figure 2 for the lower states. Results are for $\alpha_{0}=0$; that is, without ILF effect on the system ((a) panels), and $\alpha_{0}=1.0$ nm ((b) panels). The different colors indicate the number of periods calculated as indicated in Figure 3(a3). Figure 3(a1,b1) correspond to β=0, Figure 1(a2,b2) correspond to β=0.2, and finally Figure 3(a3,b3) are for β=0.5; therefore, each column corresponds to a different structure. As we can see in Figure 3(a1), the system for n=1 does not present a transmission peak; this is an approximately constant continuous function for this range of energies. This is expected behavior since, for β=0, only one potential barrier is present; the transmission is due solely to the tunneling effect. For Figure 3(a2), with n=1, the system already has two potential barriers (as can be seen in Figure 2); however, the transmission still does not reach a maximum peak for the depicted energy range. This is because, despite the existence of a resonant state in the central well region, the non-symmetry of the barriers does not allow maximum transmission of one. Furthermore, the right barrier is only 1 nm thick, so the tunneling is almost complete. Already for β=0.5 and n=1 in Figure 3(a3), the system reaches a maximum transmission value that is presented by the effect of resonant tunneling with the state inside the central well. In addition, due to the symmetry of the barriers, for a given energy value, the maximum transmission probability value is reached.

Despite the above, it should be clarified that not having a perfect resonance for the case *n* = 1 and β = 0.2 in which there is a system of non-symmetric barriers does not imply that this is true for all types of non-symmetric systems. In fact, for *n* = 2 and β = 0.2 a perfect transmission is observed, even though the system is not symmetric. Currently, there are numerous works in which the problem of perfect resonances in non-symmetric systems has been studied. These resonances can be explained in terms of local symmetries within the SL, and occur despite having a global asymmetry. Just to mention some of these works, in 2009, Nava et al. [69] studied the total transmission in Fibonacci arrays of dielectric multilayers, and found that the mirror symmetry in the SL is sufficient but not necessary for the generation of perfect resonances. Further on, Kalozoumis et al. [70] evidenced a similar behavior in one-dimensional optical media; related works can be found in the references [71,72,73].

For n=2, a well-defined peak is evidenced for the system with and without laser. In this case, the average width of the peak is greater for β=0.2 compared to β=0. Note that for β=0.5, the transmission presents a plateau-type structure, an energy range in which the transmission probability is equal to or very close to one. For n=3 and n=4, the transmission probability presents two and three peaks, respectively, for both β=0 and β=0.2. Finally, for β=0.5, the transmission probability functions present flat regions for values close to transmission equal to one. By comparing (a1–a3) with (b1–b3) in Figure 3, we note that the effect of the ILF on the system does not significantly modify the shape or average width of the electronic transmission peaks. However, there is an evident blue shift in the position of all transmission peaks for all calculated periods, independent of the value of the β-parameter. For example, the red peak in Figure 3(a1,b1), corresponding to n=2 with β=0, goes from 78.2 meV to 88 meV solely due to the laser effect. We have to highlight that the variation in the average width of each transmission peak is of fundamental importance for the response of the electric current through the device. It should be mentioned that the transmission curves presented have been calculated by means of the FEM; however, the same result is obtained by means of the transfer matrix method (TMM) [74]. A comparison is presented in Figure 3c for a GaAs-based double-well, triple-barrier system of equal well and barrier widths 5 nm; the black solid curve corresponds to the result using FEM and the red dots are obtained through TMM. Evidently, similar results are observed for both methods. Additionally, the reason why this transmission band is selected in the energy window presented is due to the fact that in the calculated range of geometric variations, there are no more quasi-stationary states inside the wells of the SL, and therefore only said transmission band is different from zero.

**Figure 4:**
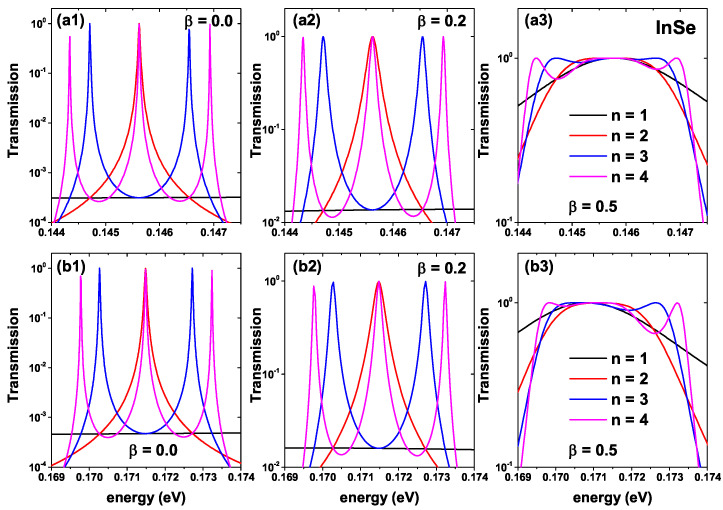
Energy dependence of the electronic transmission probabilities for an InSe-InP finite lattice, according to Figure 2, i.e., varying the *n*-number of periods and the shape according to the structural β-parameter. Results are without ILF effects (upper panels) and with $\alpha_{0}=1$ nm (lower panels). In (**a1**), $\alpha_{0}=0$ nm and β = 0.0. In (**a2**), $\alpha_{0}=0$ nm and β = 0.2. In (**a3**), $\alpha_{0}=0$ nm and β = 0.5. In (**b1**), $\alpha_{0}=1$ nm and β = 0.0. In (**b2**), $\alpha_{0}=1$ nm and β = 0.2. In (**b3**), $\alpha_{0}=1$ nm and β = 0.5.

Figure 4 shows the electronic transmission function of the well-barrier system presented in Figure 2 for the InSe-InP lattice. The upper row is for zero ILF-parameter, whereas the lower row is for $\alpha_{0}=1.0$ nm. The different colors indicate the number of calculated periods calculated in a similar way to Figure 3. The transmission functions present a trend very similar to that of the GaAs-Al${}_{0.3}$Ga${}_{0.7}$As well-barrier system; however, for the InSe-InP based system, the average width of each transmission peak for β=0 and β=0.2 presents a small but appreciable decrease concerning the GaAs-Al${}_{0.3}$Ga${}_{0.7}$As system. These differences can change the area under the transmission curve and modify the properties of electronic transport. Due to the different band offsets for both materials, clearly, the position of the maximum probability peaks is not the same. To highlight one, in Figure 3(a1) for GaAs-Al${}_{0.3}$Ga${}_{0.7}$As, the maximum probability for the system with n=1 and β=0 is given for 78.2 meV, while in the InSe-InP system with the same parameters, the resonant energy value is 145.6 meV. Finally, in Figure 4(b1–b3) we see how once again the ILF effect generates a shift towards the blue for all transmission maxima. The value of the resonance in the red curve of Figure 4(b1) appears at 171.5 meV; this indicates that the effect of the ILF on the quasi-stationary states for the InSe-InP based system is more significant than in the GaAs-Al${}_{0.3}$Ga${}_{0.7}$As one. Note that for both materials with and without laser effect, the system with β=0.5 (Figure 3(a3,b3) and Figure 4(a3,b3)) presents approximately flat regions for the transmission profile and a point at which it takes the same value regardless of the number of periods of the device. For example, for the InSe-InP based system, this value is 145.8 meV when the laser is off and 170.9 meV when the laser is on.

**Figure 5:**
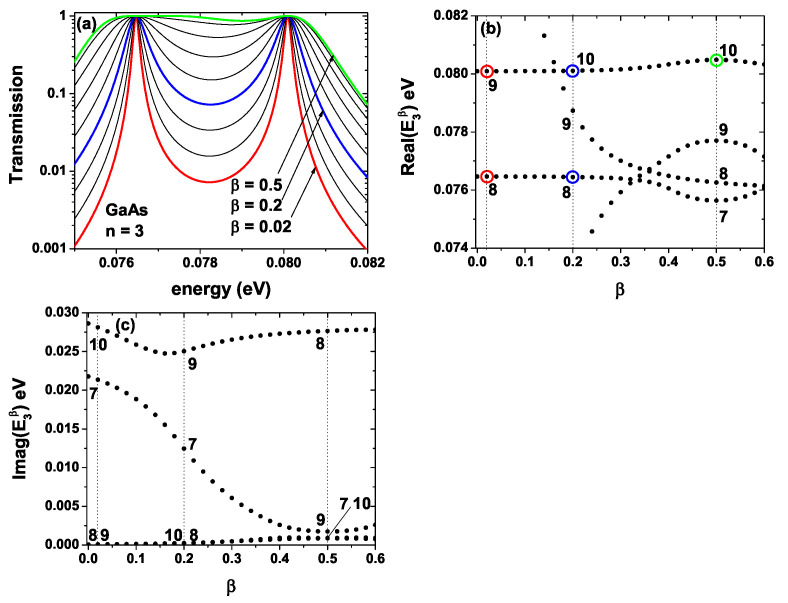
In (**a**), transmission probability for the GaAs-Al${}_{0.3}$Ga${}_{0.7}$As lattice for n=3 as a function of the electron energy for different values of the β-parameter. In (**b**,**c**), lattice eigenvalues real and imaginary parts $E_{n,i}^{\beta }$, respectively, as a function of the β-parameter.

To understand why the curves in Figure 3(a3–b3) and Figure 4(a3–b3) lose their symmetrical shape and are so different from the other figures in the rest of the panels, in Figure 5a, we have calculated the transmission probability for the GaAs-Al${}_{0.3}$Ga${}_{0.7}$As system as a function of the electron energy for n=3 and different values of the β-parameter. In Figure 5b,c, the real and imaginary parts of the lattice eigenvalues ($E_{n,i}^{\beta }$), respectively, as a function of the β-parameter, have been calculated. In Figure 5a we see that for the lowest values of the β-parameter, the transmission probability presents two clearly defined peaks, such as β=0.02 and β=0.2 highlighted in red and blue, respectively. However, as the β-value is increased, the average width of these peaks, corresponding to resonant states within the SL, increases. For β=0.5 (green curve), which is the highest value, the transmission probability has a peak around 0.08 eV and a region where the transmission probability shows an approximately flat behavior between 0.076 eV and 0.078 eV. When the Schrödinger equation is solved with open boundary conditions, a set of eigenvalues $E_{n,i}^{\beta }$ (where *i* is the order of the eigenvalue) is obtained for each pair of (n,β) values. These eigenvalues generally have real and imaginary parts. In Figure 5b, the real part of the eigenvalues for n=3 is presented as a function of the β parameter in the range (0.0,0.6). The vertical axis is in the same energy range in which the transmission probability depicted in Figure 5a has been calculated. The vertical dotted lines indicate the particular positions of β=0.02, β=0.2, and β=0.5 that correspond to the same values for the red, blue, and green curves of Figure 5a, respectively. The numbers (labels) indicate the order of the eigenvalue (*i*-parameter). The red circles in Figure 5b correspond to the value of the two eigenvalues that are presented for β=0.0; that is, these values coincide with the two maxima that are shown in the red curve of Figure 5a. As we see in Figure 5b, for β=0.2 in the range of energies presented, the system shows three eigenvalues; however, in Figure 5a the blue curve only shows two resonant peaks corresponding to the eigenvalues 8 and 10 marked by the blue circles; that is, there does not appear a to be peak associated with the eigenvalue 9 that is around 0.079 eV. The reason for this resonance not appearing can be found by analyzing Figure 5c, in which the imaginary part of the eigenvalues is presented. As we see for β=0.2, the imaginary part of eigenvalues 8 and 10 is very low compared to the imaginary part associated with eigenvalue 9. On the other hand, this imaginary part is inversely proportional to the average lifetime of the electrons in each state inside each well. From the above, it is obtained that the lifetime of the electrons in state 9 is very low compared to states 8 and 10, which implies a probability density equal to zero at the interior of the wells of the SL for this state (this energy does not coincide with a quasi-state inside the wells), which disables it for the resonant tunneling process, and for this reason, that third peak does not appear in the blue curve of Figure 5a.

Analogous behavior happens for β=0.5; in Figure 5b, we see that four eigenvalues appear in the range of energies presented; three of them very close between 0.075 eV and 0.078 eV and one more close to 0.08 eV. When we analyze the imaginary part of each of these states by means of Figure 5c, it is found that the imaginary part associated with state 8 is very large compared to the other states for this value of β. Additionally, the probability density associated with this state is zero inside the wells; in other words, this energy does not correspond to a quasi-steady state. From the above, it is concluded that the green curve in Figure 5a corresponds to the contribution of states 7, 9, and 10, where state 10, highlighted in Figure 5b with a green circle, corresponds to the resonance around 0.08 eV, as shown in Figure 5a.

The geometry modifications of the system, particularly for large values of β, generate an increase in the barrier further to the right of the SL. This fact allows the emergence of an additional state inside the system, generating a transmission profile that passes off clear resonances towards a band structure. From these results, we can say that the plateaus on the green curve are caused by an increase in the width of the resonant peaks at the same time as a superposition of three states, when initially for small β, there were only two states. It is worth mentioning that similar behavior was evidenced by Barra and Gaspard in the spectrum of scattering resonances in spatially extended open-quantum systems [75].

**Figure 6:**
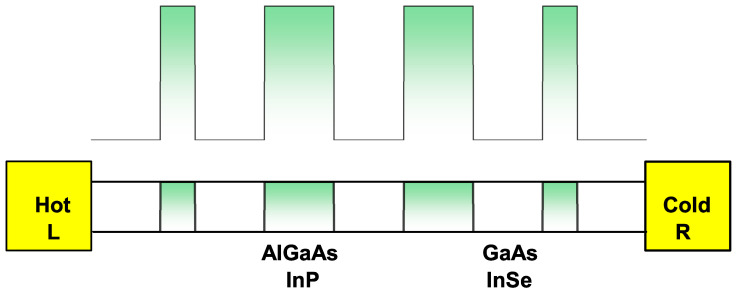
Schematic diagram of the SL made up of a system of GaAs (InSe) wells and Al${}_{0.3}$Ga${}_{0.7}$As (InP) barriers. The lower part represents the device connected to hot-left and cold-right reservoirs that also act as emitters and collectors. The top represents the bottom of the conduction band of the system. The width of the wells and the two central barriers have been set at 5 nm, and the barriers at the left and right are set at 2.5 nm (see Figure 2(c3)).

**Figure 7:**
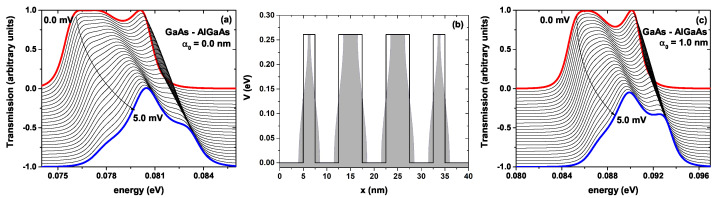
Corresponds to the system shown in Figure 2(c3). (**a**) the transmission probability for the GaAs-Al${}_{0.3}$Ga${}_{0.7}$As lattice without laser effect for different bias voltages concerning the incident electron energy; (**b**) the scheme of the potential profile for the barrier-well lattice system; the black curve corresponds to the system without laser effect and the shaded region including an ILF parameter strength of $\alpha_{0}=1.0$ nm; (**c**) the same as in (**a**), but with an ILF effect, $\alpha_{0}=1.0$ nm. The width of the wells and the two central barriers have been set at 5 nm and the two lateral barriers at 2.5 nm.

**Figure 8:**
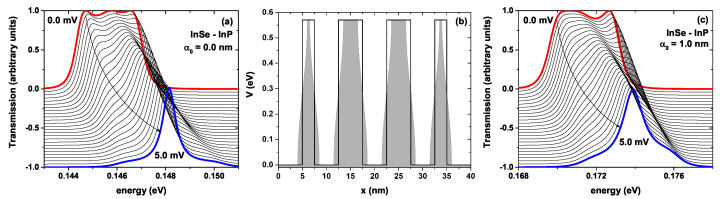
In (**a**), the transmission probability for the InSe-InP lattice without laser effect for different bias voltages concerning the incident electron energy; (**b**) the scheme of the potential profile for the barrier-well lattice system; the black curve corresponds to the system without laser effect and the shaded region including an ILF parameter strength of $\alpha_{0}=1.0$ nm; (**c**) the same as in (**a**), but with an ILF effect, $\alpha_{0}=1.0$ nm. The width of the wells and the two central barriers have been set at 5 nm and the two lateral barriers at 2.5 nm.

Figure 6 shows the schematic diagram of the SL made up of a system of GaAs (InSe) wells and Al${}_{0.3}$Ga${}_{0.7}$As (InP) barriers. The lower part represents the device connected to two (hot and cold) reservoirs. The top represents the bottom of the conduction band of the system. The width of the wells and the two central barriers have been set at 5 nm and the end barriers at 2.5 nm. The system can be brought out of equilibrium through a potential or temperature difference between the electronic reservoirs. This would induce a flow of the charge carriers, which implies an electric or thermal current between the device terminals. An external ILF can be applied to this well-barrier arrangement and can analyze how the electronic probability changes; this is represented in Figure 7 and Figure 8.

Figure 7 shows the transmission probability for the GaAs-Al${}_{0.3}$Ga${}_{0.7}$As system without ILF effect for different bias voltages concerning the incident electron energy, Figure 7a. Figure 7b shows the scheme of the potential profile for the barrier-well lattice system—see Figure 2(c3)—where the black curve corresponds to $\alpha_{0}=0$ and the shaded region for $\alpha_{0}=1.0$ nm. In Figure 7c, it is depicted the same as in Figure 7a, but for $\alpha_{0}=1.0$ nm. In Figure 7a, we can see how as the bias voltage increases, the flat peak at the left side becomes narrower; that is, more defined, and moves towards higher energies; this can be seen by comparing the corresponding red curve at zero bias—see Figure 3(a3) blue curve—with the blue curve corresponding to 5.0 mV. Additionally, it is observed that the peak located at higher energies loses intensity in a systematic way as the bias voltage increases. In summary, at zero bias voltage, the transmission probability presents two structures, a flat low-energy structure and a narrow and well-defined high-energy structure, which collapse into a single, much more defined structure with a probability of 1.0 and localized at 80.5 meV when the bias voltage is 5.0 mV. According to Landauer’s theory, the area under each probability curve must be proportional to the electric current due to the flow of electrons through the system for this voltage. For the calculations presented in Figure 7a, the profile of the bottom of the conduction band shown in Figure 7b (black curve) has been used. Figure 7c shows the transmission probability for the GaAs-Al${}_{0.3}$Ga${}_{0.7}$As system calculated for the potential profile of Figure 7b (shaded region) including the ILF effects. As we see in Figure 7b, the non-resonant ILF induces a decrease in the width of the well-bottom; this causes the quasi-stationary levels to rise, which corresponds to a blue shift, as evidenced in Figure 7c. In Figure 7c, the zero bias curve corresponds to the Figure 3(b3) blue plot, and shows similar behavior to that presented in Figure 7a as the bias voltage is increased in the system; that is, there is a decrease in the intensity of the extreme peaks and the emergence of a single central peak of maximum probability (one) attached to two external peaks of less intensity. However, there is clearly a notable difference in the area under each curve compared to the ILF effects as the voltage increases. These differences in transmission probability profiles cause changes in electronic transport properties.

The results depicted in Figure 8 follow the same scheme as Figure 7, but for the InSe-InP system. In Figure 8a, we can see how the system goes from having three peaks all associated with maximum probability 1, the red curve for zero bias voltage (see Figure 4(a3) without laser effect and Figure 4(b3) with intense laser effects) having a single central peak with the probability of one and two shoulders of smaller amplitude on each side (blue curve). If we compare Figure 7a and Figure 8a, we see that for both materials, there is a blue shift in the position of the transmission peaks with the increase in voltage; however, the average width of the peaks is greater in the case of the GaAs-Al${}_{0.3}$Ga${}_{0.7}$As structure than in the InSe-InP one. Analogous behavior occurs when an intense non-resonant laser is applied to the system; see Figure 7c and Figure 8c. From Figure 7 and Figure 8, it has been found that the transmission profile of the well-barrier lattice system can be modified by applying an external non-resonant laser field and modifying the materials that make up each layer of the system, and of course, applying a bias voltage between the emitter and collector, maintaining fixed geometric parameters. This indicates that this system is a good candidate for an electronic device, since it is possible to tune the electronic transmission through which physical quantities such as electric current, heat flow, conductance, and power, among others, can be modeled.

**Figure 9:**
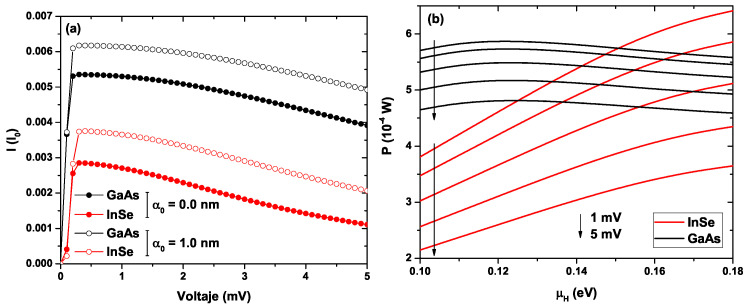
In (**a**), the electronic tunneling current for the well-barrier lattice system as a function of the bias voltage, in units of $I_{0}=2\;e/h$, for zero ILF-parameter (full symbols) and $\alpha_{0}=1.0$ nm (open symbols). In (**b**), the power for five different bias voltages concerning the chemical potential of the hot reservoir. The width of the wells and the two central barriers has been set at 5 nm and the two lateral barriers at 2.5 nm (see Figure 2(c3)). Calculations are for GaAs-Al${}_{0.3}$Ga${}_{0.7}$As and InSe-InP lattices.

Figure 9a shows the electronic tunneling current for the well-barrier lattice system as a function of the bias voltage. Analyzing the current for both materials without including laser effects (full symbols), it is found that the system based on GaAs-Al${}_{0.3}$Ga${}_{0.7}$As reaches a value of 5.4 m$I_{0}$ for the maximum current peak that occurs at 0.3 mV, and on the other hand, for InSe-InP-based material, the maximum current value is 2.9 m$I_{0}$, which occurs for the same bias voltage. It should be noted that, for both materials, the geometric parameters have remained unchanged. Both materials present an abrupt increase in the current between zero and 0.3 mV to later present a monotonous decreasing behavior, reaching up to 3.9 m$I_{0}$ for GaAs-Al${}_{0.3}$Ga${}_{0.7}$As and up to 1.1 m$I_{0}$ for InSe-InP. These differences between the currents of both materials are due to the differences in the heights of the potential barriers generated by the different band offsets. The above generates changes in the area under the curve of the electronic transmission probability, which can be evidenced by comparing Figure 7a and Figure 8a, where we see that the area under the curve is greater for the GaAs-Al${}_{0.3}$Ga${}_{0.7}$As system and according to Landauer’s theory, the tunneling current is proportional to this area. With the application of the ILF ($\alpha_{0}=1.0$ nm), as found in previous figures, there is a shift in the quasi-stationary states inside the wells; in the same way, there is a small increase in the area under the curve of electronic transmission for both materials. This fact implies that the tunneling current also shows an increase. For the system based on GaAs-Al${}_{0.3}$Ga${}_{0.7}$As, the maximum current peak is now 6.2 m$I_{0}$ at 0.3 mV and that of InSe-InP is 3.8 m$I_{0}$ at 0.4 mV. The behavior is similar to the system without ILF effects, presenting an abrupt increase for low voltages and a subsequent decrease. Lattices based on GaAs-Al${}_{0.3}$Ga${}_{0.7}$As and InSe-InP reach the values of 4.9 m$I_{0}$ and 2.1 m$I_{0}$, respectively, for 5 mV. This indicates that both by changing the materials and through the application of an external laser field, it is possible to modify the properties of electronic transport, such as the electric current in the wells and barriers system. It should be noted that, for both materials, a NDR effect appears, which can be controlled in magnitude with the external laser field. This generates a great advantage when it comes to implementation in a device, since it is possible to control the current–voltage characteristics in the heterostructure without the need to make geometric changes that are much more complex from the experimental point of view.

Figure 9b shows the power for the GaAs-Al${}_{0.3}$Ga${}_{0.7}$As system (black curves) and InSe-InP (red curves) for five different bias voltages concerning the chemical potential of the hot reservoir. The GaAs system shows close to flat power curves, having an increasing slope for low values of the chemical potential, going through a maximum, and decreasing slowly. The InSe system shows almost a lineal behavior, starting from low chemical potential values and increasing to larger magnitudes. As the bias voltage is increased, the value of the power in each system decreases. On the other hand, for 1 mV, the power is maximum for the GaAs-Al${}_{0.3}$Ga${}_{0.7}$As system only for $\mu_{H}<0.15$ eV, since after this value, the power is now maximum in the InSe-InP system. This indicates that for the chemical potential of the hot reservoir at 0.15 eV, both materials present the same power value of 5.8 ($10^{-4}$ W) for a bias voltage of 1 mV; similarly, each crossing of the red curves with the black ones indicates points in which the power is equivalent for both materials and at different bias voltages.

When the potential difference between the terminals of the system is increased, an additional asymmetry in the potential profile is generated, which leads to a decrease in the probability of electronic transmission (area under the curve in the band of transmission) as evidenced in Figure 7 and Figure 8. This behavior also occurs when the system is subjected to the effect of an intense non-resonant laser field. This decrease in transmission implies a decrease in the tunneling current that is evidenced through the Landauer formalism and the result presented in Figure 9a. This behavior can also be understood as an increase in the electric current when the energy of the incident electrons (coming from the emitter) coincides with the energy of the quasi-stationary states inside the SL; in this case, these states are known as resonant states. When this resonance does not exist, there is a decrease in the electric current. There is a relationship between NDR and negative differential conductance (NDC), since both transport properties can be studied from the Landauer formalism and the physical origin of both phenomena is identical; that is, a decrease in electrical current generated from an increase in external voltage. More clearly referring to the relations $G_{diff}=1/R_{diff}=dI/dV$, where $G_{diff}$ is the NDC, $R_{diff}$ is the NDR, *I* is the current, and *V* is the applied voltage. This effect not only arises in semiconductor systems; it also occurs in molecular systems; see Refs. [76,77,78].

## 4. Conclusions

The finite SL systems based on GaAs-Al${}_{0.3}$Ga${}_{0.7}$ and InSe-InP have been studied by analyzing how the probabilities of electronic transmission change for both materials by varying the geometric parameters that allow changes in the number of periods in the lattice, as well as the well and barrier widths. By applying an external non-resonant ILF, it is possible to modify the lattice potential profile, which leads to a blue shift in quasi-stationary states. A four-barriers-three-wells arrangement was undertaken to analyze the electronic transport properties by means of Landauer’s theory for both semiconductor materials, including the laser effects. It was found that, for the same geometry, the tunneling current was higher for the GaAs-Al${}_{0.3}$Ga${}_{0.7}$As based system than that of InSe-InP based system; in the same way, the point of maximum current was practically unchanged for both materials for the calculated bias voltages. By applying the external laser field, an increase in the tunneling current was found for both materials but maintained the same trend of the I-V curves. For both materials, a NDR effect appeared that can be controlled in magnitude with the external laser field. This generates a great advantage when it comes to implementation in a device, since it is possible to control the current–voltage characteristics in the heterostructure without the need to make geometric changes that are much more complex from the experimental point of view.

Finally, the power for the same arrangement was calculated concerning the chemical potential, finding higher values for the system based on GaAs-Al${}_{0.3}$Ga${}_{0.7}$As for small values of the chemical potential. For high values, the power was more significant in the InSe-InP system. It should be noted that, for a set of points, the power for both systems took the same value. A four-barriers-three-wells arrangement was undertaken to analyze the electronic transport properties by means of Landauer’s theory for both semiconductor materials, including the laser effects. It was found that, for the same geometry, the tunneling current was higher for the GaAs-Al${}_{0.3}$Ga${}_{0.7}$As based system than for the InSe-InP based system. In the same way, the point of maximum current was practically unchanged for both materials for the calculated bias voltage. It is worth mentioning that high harmonic multipliers are among the most recent applications of SL based on semiconductor materials. Considering the geometric modifications analyzed in this work, it is possible to characterize these systems that are widely studied today.

## Figures and Tables

**Table 1:** Geometric parameters for different values of β.

β	*a* (nm)	*w* (nm)	$b_{1}$ (nm)	$b_{2}$ (nm)
0.0	10	5	5	0
0.2	10	5	4	1
0.5	10	5	2.5	2.5

## Data Availability

No new data were created or analyzed in this study. Data sharing is not applicable to this article.
